# Critical role of FOXO3a in carcinogenesis

**DOI:** 10.1186/s12943-018-0856-3

**Published:** 2018-07-25

**Authors:** Ying Liu, Xiang Ao, Wei Ding, Murugavel Ponnusamy, Wei Wu, Xiaodan Hao, Wanpeng Yu, Yifei Wang, Peifeng Li, Jianxun Wang

**Affiliations:** 1 Institute for Translational Medicine, College of Medicine, Qingdao University, Qingdao, 266021 China; 2 Department of comprehensive internal medicine, Affiliated Hospital, Qingdao University, Qingdao, 266003 China

**Keywords:** FOXO3a, Tumor suppressor, Post-translational modifications, Inactivation, Cancer

## Abstract

FOXO3a is a member of the FOXO subfamily of forkhead transcription factors that mediate a variety of cellular processes including apoptosis, proliferation, cell cycle progression, DNA damage and tumorigenesis. It also responds to several cellular stresses such as UV irradiation and oxidative stress. The function of FOXO3a is regulated by a complex network of processes, including post-transcriptional suppression by microRNAs (miRNAs), post-translational modifications (PTMs) and protein–protein interactions. FOXO3a is widely implicated in a variety of diseases, particularly in malignancy of breast, liver, colon, prostate, bladder, and nasopharyngeal cancers. Emerging evidences indicate that FOXO3a acts as a tumor suppressor in cancer. FOXO3a is frequently inactivated in cancer cell lines by mutation of the FOXO3a gene or cytoplasmic sequestration of FOXO3a protein. And its inactivation is associated with the initiation and progression of cancer. In experimental studies, overexpression of FOXO3a inhibits the proliferation, tumorigenic potential, and invasiveness of cancer cells, while silencing of FOXO3a results in marked attenuation in protection against tumorigenesis. The role of FOXO3a in both normal physiology as well as in cancer development have presented a great challenge to formulating an effective therapeutic strategy for cancer. In this review, we summarize the recent findings and overview of the current understanding of the influence of FOXO3a in cancer development and progression.

## Background

Forkhead box (FOX) proteins are evolutionarily conserved transcription factor family of proteins, which are characterized by their forkhead winged helix-turn-helix DNA binding domain composed of three α–helices and two loop or “wing” domains. Currently, more than 2000 members have been found in this family of transcription factors based on sequence homology, which are ubiquitously expressed across a range of species from yeast to human [1, 2]. FOX proteins regulate a wide spectrum of biological processes involved in normal homeostasis and development [3, 4]. Although the forkhead DNA binding domain with ~ 100 amino acid residues is highly conserved, the other domains are very divergent in FOX proteins. So they have very different binding specificities and cellular effects. According to additional domains and sequence conservation, FOX family is further grouped into various subfamilies, namely FOXM, FOXK, FOXA and FOXO families [5–7].

The forkhead box class O (FOXO) family is a ubiquitously expressed transcription factor that plays important role in higher organisms. The first member of this family with *fork head* was described in *Drosophila*, which plays key roles in the terminal development of Drosophila embryo [8]. The mammalian system consists of four members, namely FOXO1, FOXO3a, FOXO4, and FOXO6, which are known to be regulated by the phosphoinositol-3-kinase (PI3K)-PKB signaling pathway [9–11]. FOXO family has been shown to regulate developmental processes and energy metabolism as well as tumorigenesis in many tissues. All these functions are mediated by the specific activation of a coordinated transcriptional program [12]. The deregulation of FOXO functions will cause uncontrolled cell proliferation and accumulation of DNA damage, which results in carcinogenesis.

The member of FOXO subfamily, FOXO3a, also known as FOXO3 or forehead in rhabdomyosarcoma-like 1 (FKHRL1), was first identified in human placental cosmid. The *FOXO3a* gene is located on chromosome 6q21 [13] and it plays vital role in regulating a variety of cellular processes through targeting the expression and activity of effector genes. The subcellular localization of FOXO3a is important for its activities and functions [14]. The phosphorylation of FOXO3a leads to its translocation from nucleus to cytoplasm, where it associates with 14–3-3 protein and this binding prevents its reentry into the nucleus [15, 16]. In this review, we focus on the recent findings and important progress made in identification of FOXO3a functions and its target molecules and we have also presented an overview of the current understanding of the influence of FOXO3a activity on cancer.

## Overview: Structure, regulation and function of FOXO3a

### Structural domains of FOXO3a

**Fig. 1:**
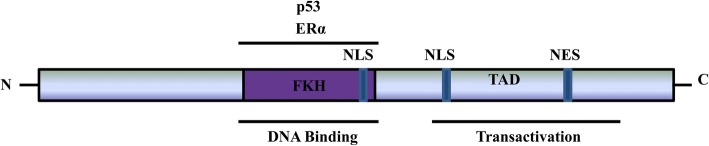
Structure of human FOXO3a. Letters within the bar indicate structural domains. The well-known proteins interacting with FOXO3a are shown above the lines at the corresponding domains. Only representatives of FOXO3a-interacting proteins are shown. FKH, forkhead winged helix-turn-helix DNA binding domain; TAD, transactivation domain; NLS, nuclear localization sequence; NES, nuclear export sequence

FOXO3a is approximately 71 kDa in size and its structure is conserved across different species. FOXO3a contains five domains: a highly conserved forkhead winged helix-turn-helix DNA binding domain (FKH), two nuclear localization sequence (NLS), a nuclear export sequence (NES) and C-terminal transactivation domain (TAD) (Fig. 1). Among the FOXO family members, many of these regions are highly conserved. A highly conserved Forkhead Domain is primarily responsible for direct interaction between FOXO3a and DNA, which also mediates its interaction with Estrogen receptor α (ERα) [17] and p53 [18]. NLS domain is required for the translocation of FOXO3a from cytoplasm to nucleus and it also mediates the release of FOXO3a from nucleus [19]. TAD domain in C-terminal is vital for the transactivation of FOXO3a target genes.

### Regulation of FOXO3a activity

#### MiRNA pathways contribute to post-transcriptional regulation of FOXO3a

MicroRNA (miRNA) is a kind of short single-stranded non-protein-coding RNA molecules that negatively regulates the gene expression at the posttranscriptional level by repressing translation and/or promoting mRNA degradation [20, 21]. There are more than 30% of genes are regulated by miRNA in human system [22]. The 3′-untranslated region (3′-UTR) of FOXO3a mRNA harbors several miRNA target sequences. Many miRNAs modulate the expression of FOXO3a proteins under various pathological conditions. *FOXO3a* is directly targeted by miR-155 in ischemic renal diseases and some types of cancer. Experimental studies revealed that the overexpression of miR-155 down-regulates the expression of FOXO3a protein, while knockdown of miR-155 increases FOXO3a expression [23–27]. FOXO3a is also regulated by other miRNAs, including miR-132, miR-212 and miR-223. They directly bind to FOXO3a 3′-UTR and inhibit the expression of FOXO3a. The de-repression of FOXO3a by microRNA-132 and 212 cause neuronal apoptosis in Alzheimer’s disease [28]. In addition, miR-132 and 223 promote pathogenesis of inflammatory bowel disease by negatively regulating FOXO3a [29]. In glioblastoma cells, the overexpression of miR-27a can inhibit the expression of FOXO3a protein and its transcriptional activity, while the inhibition of miR-27a increases the expression and activity of FOXO3a, which indicates that FOXO3a is a target of miR-27a [30]. In traumatic brain injury condition, miR-27a displays neuroprotective effect by directly targeting FOXO3a-mediated neuronal autophagy [31]. In human breast cancer and Idiopathic pulmonary fibrosis, miR-96 directly targets the 3′UTR of the FOXO3a mRNA, which consequently decreases the expression of FOXO3a targets (p27 and p21) and increasing cyclin D1 [32, 33]. FOXO3a can also be directly regulated by other miRNAs, such as miR-30d, miR-182, miR-592, miR-1307 and 29a [34–38]. Modulation of FOXO3a by anti-miR strategies may prove useful to promote apoptosis. In addition to the direct regulation of miRNA, FOXO3a activity also can be regulated by miRNAs in an indirect manner. For instance, miR205 upregulates AKT dependent activation of FOXO3a in lung cancer cell via suppressing PTEN [39]. Therefore, to explore the comprehensive of network of microRNAs and FOXO3a, further research is required to design FOXO3a based strategies for better chemotherapeutics.

#### Importance of post-translational modifications in regulation of FOXO3a

Post-translational modifications (PTMs) is the fundamental process for the regulation of proteins’ functions that cause changes in their subcellular location, molecular half-life, DNA-binding affinity and/or interaction with other cellular proteins. The common PTMs include phosphorylation, acetylation, methylation, ubiquitination, sumoylation, neddylation, glycosylation, sulphation and prenylation. The activity of FOXO3a can be regulated by multiple types of PTMs including phosphorylation, acetylation, ubiquitination and methylation [9, 40, 41]. These reversible PTMs alter the translocation of FOXO3a, influence its DNA binding affinity, and change the pattern of transcriptional activity at specific target genes sites [42, 43]. These modifications in FOXO3a occur consecutively by various combinations of enzymes and signaling molecules.

The primary mechanism of regulation of FOXO3a activity and its target genes is by controlling the translocation of FOXO3a between nucleus and cytoplasm, which can be achieved by phosphorylation by a series of kinases. The protein kinases such as protein kinase B (PKB), extracellular signal-regulated kinase (ERK), Serum-and glucocorticoid-inducible kinases (SGK) and IκB kinase isoform β (IKKβ) promote the nuclear export of FOXO3a [44–47]. Whereas, poly(ADP-ribosyl)ated by PARP1 dependent phosphorylation facilitates its exclusion from the nucleus [48]. After the cytoplasmic retention, FOXO3a is ubiquitinated and then degradated by proteasome [45]. The sites for PTMs in FOXO3a is well defined and activation of these kinases normally correlates with loss of nuclear FOXO3a. However, the phosphorylation of FOXO3a by p38, Macrophage stimulating 1 (MST1) and AMPK promote its nuclear entry and increase its transcriptional activity [49–51]. Given the fact that the balancing of nuclear import and export is very important to maintain FOXO3a functions, the loss of this balance leads to development and progression of various diseases including cancer.

The PTMs of nuclear FOXO3a regulates its transcriptional activity by changing DNA binding affinity and promoter binding specificity. In nucleus, FOXO3a is acetylated by p300 and CREB-binding protein (CBP) and it is deacetylated by SIRT1 and SIRT2. Interestingly, SIRT1 mediated deacetylation changes the DNA binding affinity of FOXO3a [52], while deacetylation by SIRT2 increases its DNA-binding activity [53]. The coactivator-associated arginine methyltransferase 1 (CARM1) dependent methylation of FOXO3a is required for its activation in the nucleus [40]. A molecular study found that the methylation of FOXO3a at K270 leads to the loss of DNA binding ability and it reduces FOXO3a-mediated apoptosis. Many PTMs of FOXO3a can interact with each other, and function in combination or compete with each other. Therefore, exploring the FOXO code is essential to understand the function and mechanism of FOXO3a.

#### Alternative protein–protein interactions modulate FOXO3a activity

The activity of FOXO3a can be modulated by other proteins via protein-protein interactions. As a transcription factor, FOXO3a interacts with co-regulators (co-activators or co-repressors) and general transcription factors to regulate the gene expression of its target. In neuronal cells, C/EBP homologous protein (CHOP) directly interacts with FOXO3a in response to endoplasmic reticulum stress and that increases the transcription activity of FOXO3a and inducing the expression of FOXO3a target genes *Puma* and *Bim* [54]. In many cancer cell lines, c-Myc binds with FOXO3a and this interaction represses FOXO3a-mediated activation of the *p27* promoter as evident from consistent with the inverse patterns of their expression in a diverse group of human cancers [55]. In MCF-7 cells, latency associated nuclear antigen 2 (LANA2) functionally interacts with FOXO3a and inhibits the transactivation of *Bim* promoter mediated by FOXO3a [56]. In normal lympho-blasts and HeLa cells treated with H_2_O_2_, forms a complex with FOXO3a by direct binding with FANCD2 in response to oxidative stress [57]. In COS-7 cells, the interaction of p53 with FOXO3a suppresses transcriptional activity of FOXO3a. In fact, p53 decreases the expression of apoptosis-inducible genes such as *Bim* and *Bcl6*, but it does not affect the expression of *p27* and *Cyclin G2* [58]. In HeLa cells, FOXO3a is de-phosphorylated by PP2A interaction, which results in the rapid nuclear translocation and transcriptional activation of FOXO3a [59]. In Gastric Cancer Cells, the complex of RUNX3 and FOXO3a participates in the induction of apoptosis by activating FOXO3a target gene *Bim* [60]. In the Mitochondria, the interaction of SIRT3 with FOXO3a increases FOXO3a DNA-binding activity as well as FOXO3a dependent gene expression [61].

### Functions of FOXO3a

**Fig. 2:**
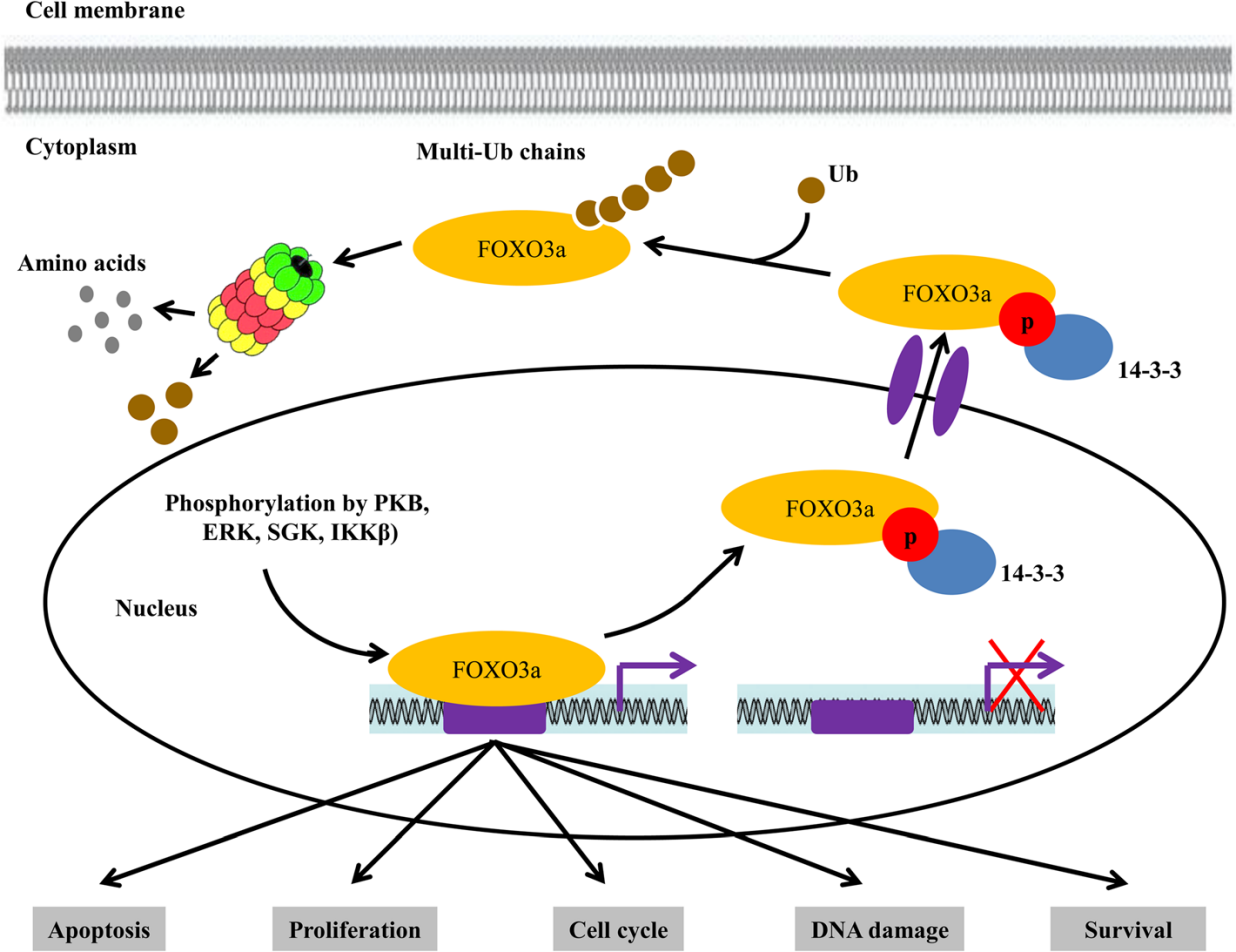
The functions and regulation of FOXO3a. The non-phosphorylated form of FOXO3a located in nucleus actively mediates multiple cellular processes, including cell apoptosis, proliferation, cell cycle, survival and DNA damage by inducing transcription of its target genes depends on the upstream stimuli. The growth factor signaling induced activation of protein kinases such as PKB, ERK, SGK, IKKΒ terminate FOXO3a activity by phosphorylation (in active form). The phosphorylated FOXO3a binds with 14–3-3 protein, which consequently leads to nuclear export of FOXO3a. In the cytoplasm, FOXO3a is ubiquitinated and degraded in a proteasome-dependent manner

FOXO3a is a central transcription factor that mediates multiple physiological and pathological processes by inducing transcription of target genes involved in apoptosis [62], proliferation [63], cell cycle progression [64], survival [65] and DNA damage [66] (Fig. 2). It also respond to several cellular stresses such as UV irradiation [67] and oxidative stress [68, 69]. Besides, FOXO3a is strongly associated with human longevity [70]. FOXO3a is also involved in the regulation of autophagy process in muscle and in cancer cells [71, 72]. The multiple functions of FOXO3a indicate that deregulation of FOXO3a expression and/or activity can lead to various diseases, particularly cancer. Indeed, the overexpression of FOXO3a has been shown to inhibit tumorigenesis in breast cancer [17, 73]. The export of FOXO3a from nucleus seems to be related to poor survival of breast cancer patients [73]. In this context, the tumor suppressor function of FOXO3a is also well defined in other type of cancers.

## FOXO3a in diseases development

### FOXO3a and its role in non-neoplastic diseases

The dysregulation of FOXO3a has been implicated in many pathological processes. FOXO3a play a crucial role in neurological disorders such as Alzheimer’s diseases, Lewy body dementia, Parkinson’s diseases, motor neuron disease and acute spinal cord injury. FOXO3a also associated with the development of heart disease, muscle atrophy, and premature ovarian failure.

Alzheimer’s disease (AD) is a most common form of age-associated dementia, which is a multifactorial and progressive neurodegenerative disorder. The mRNA and protein levels of FOXO3a are significantly up-regulated, and most of the its target genes are increased in AD brains, which indicates that the FOXO3a signaling pathway contributes to AD neurodegeneration [28]. In the Tg2576 mouse model of AD, the inactivation of FOXO3a had attenuated AD-type amyloid neuropathology. In primary neuron cultures derived from Tg2576 mouse embryos, a constitutively active form of FOXO3a promotes AD amyloid-β peptide (Aβ) levels by inhibiting non-amyloidogenic α-secretase activity, which indicates the existence of an inverse correlation between FOXO3a activity and Q Aβ amyloidosis [74].

Parkinson’s disease (PD) and Lewy body dementia (LBD) are recognized as disorders of protein aggregation and inclusion body formation. The increased activity and expression of FOXO3a is intimately associated with Lewy bodies and Lewy neurites in the brain tissue of LBD and PD. In fact, the localization of FOXO3a to Lewy bodies result in the degeneration of neurons [75].

The cardiovascular problems and its associated complications are the leading cause of mortality worldwide. FOXO3a acts as a negative regulator of cardiomyocyte size in the cardiac tissue [76]. Our previous study demonstrated that FOXO3a inhibits cardiomyocyte hypertrophy by transcriptionally targeting catalase [77]. In pathological hypertrophy and heart failure, FOXO3a drives the expression of BNIP3 and induces mitochondrial apoptosis and mitophagy [78]. FOXO3a can inhibit cardiomyocyte hypertrophy by suppressing the expression of *p21*, *Cat* and *Atrogin-1* [77, 79, 80], which are involved in hypertrophic response.

Recent studies demonstrate that FOXO3a up-regulates the expression of the atrophy-related ubiquitin ligases atrogin-1 and muscle Ring Finger-1, which induce a rapid loss of muscle mass [81, 82]. Hsp70 and SAPKs inhibit the activity of FOXO3a and prevent skeletal muscle atrophy [83, 84]. On the other hand, FOXO3a promotes cell survival pathway in aortic vascular smooth muscle cells. However, its deregulation due to a reduction of IGF-1R signaling may promote apoptosis during atherosclerosis [85]. FOXO3a is a critical regulator of follicular activation. A study in mice with ovarian phenotype of *FOXO3a*^*−/−*^showed a similar phenotype with the human premature ovarian failure (POF). A mutation screening in POF patients have revealed that there are eight variants in *FOXO3a* and three of them are resulting in amino acid substitutions, which indicates that *FOXO3a* is a candidate gene for POF in human [86]. After acute spinal cord contusion injury, a significant decrease in the expression of FOXO3a favors axonal regeneration and glial cell proliferation by reduction in the expression of its target protein p27^kip1^, which indicates that FOXO3a has a detrimental role in nervous system lesion and repair [87]. In contrast, the pharmacological or genetic activation of FOXO3a protects neurons from damage caused by motor neuron diseases [88].

### Implication of FOXO3a in carcinogenesis

It is well known that FOXO3a has a crucial role in apoptosis, cell proliferation, DNA damage and resistance to oxidative stress, and thus its deregulation of FOXO3a is highly associated with a series of malignancies [60, 89–103] (Table 1). In most of the malignant cells, the deregulation of FOXO3a is mainly through aberrant PTMs.

**Table 1:** Functional roles of FOXO3a pathway in different types of cancer

Cancer types	Key message(s)	Ref.
Breast cancer	Dephosphorylation of FOXO3a induced by Aplysin suppresses tumor growth by inhibiting cell proliferation and promoting apoptosis in cancer cells.	[89]
Prostate cancer	Deregulation of FOXO3a promotes prostate cancer progression in TRAMP mice.	[90]
Acute myeloid leukemia	Dephosphorylation of FOXO3a induced by hypomethylating agents promote apoptosis by upregulation of BIM and PUMA expression.	[91]
Colon cancer	Activation of FOXO3a by aldose reductase induces human colon cancer cell apoptosis by upregulating both DR5 and DR4.	[92]
Lung cancer	Deregulation of FOXO3a promotes DNMT3B overexpression leading to tumor growth in lung cancer.	[93]
Glioma	A high expression of FOXO3a is associated with glioblastoma progression and FOXO3a level independently indicates poor prognosis in Glioma patients.	[94]
Thyroid cancer	Nuclear FOXO3a promotes cell cycle progression by transcriptional upregulation of cyclin A1 and accelerates proliferation of human ATC cells.	[95]
Lung adenocarcinoma	FOXO3a gene inactivation occurs frequently in carcinogen-induced lung adenocarcinoma.	[96]
Oral squamous cell carcinoma	Constitutively active form of FOXO3a induces significant G1-phase arrest and apoptosis in OSCC cells	[97]
Neck cancer	Tumor patients with low FOXO3a expression have a poor prognosis compared with patients with high FOXO3a.	[98]
Urothelial cancer	FOXO3a suppresses invasiveness of urothelial cancer through regulation of Twist1, YB-1 and E-cadherin.	[99]
Osteosarcoma	Activation of FOXO3a by ionizing radiation induces cell apoptosis in osteosarcoma.	[100]
Bladder cancer	Upregulation of FOXO3a by Nkx2.8 suppresses bladder cancer proliferation.	
Gastric cancer	FOXO3a cooperates with RUNX3 to induce apoptosis by activating *Bim* in gastric cancer cells.	[60, 101]
Neuroblastoma	Inactivation of FOXO3a by AKT is essential for neuroblastoma cell survival.	[102]
Ovarian cancer	Inhibition of FOXO3a phosphorylation by BrMC upregulates Bim expression and leads to apoptosis in ovarian cancer cells.	[103]

#### Deregulation of FOXO3a phosphorylation

FOXO3a is phosphorylated by several upstream kinases, such as Akt, ERK, SGK, IKKβ and IKBKE [104]. The phosphorylated FOXO3a is expelled from nucleus by binding with 14–3-3 proteins and through exportins. In the cytoplasm, FOXO3a is further ubiquitinated and then degradated by an ubiquitin/proteasome-dependent manner [105]. The deregulation of these kinases are frequently observed in different kinds of cancers and that contributes to the progression of carcinogenesis by promoting the nuclear-to-cytoplasm translocation and/or ubiquitin/proteasome dependent degradation of FOXO [106].

The role of JNK in cancer is still in debate that has pro-oncogenic as well as tumor-suppressor roles in cancer tissue depends on the upstream signaling. Its expression and/or activity is dysregulated during carcinogenesis [107]. The abnormal activation of JNK by UV irradiation inactivates ERK and PKB, which, in turn, leads to cell death by increased activity of nuclear FOXO3a and Bim expression [67]. IKK plays important roles in chromatin remodeling, cell cycle progression and nuclear factor κB (NFκB) signaling pathway, which is involved in the development of disorders, including cancer [108]. IKK directly interacts with and phosphorylates FOXO3a independent of PKB, and that causes the degradation of FOXO3a. The cytoplasmic level of FOXO3a correlates with the expression of IKKβ in many types of tumor. The negative regulation of FOXO3a by IKK plays a key role in promoting malignant cell growth and tumorigenesis [73]. The RAS–ERK signaling pathway can be activated by a wide range of extracellular growth signals that is known to play a crucial role in differentiation, proliferation and tumor progression. A constitutively active ERK phosphorylates FOXO3a and consequently promotes its degradation, thereby ERK pathway contributing to carcinogenesis [45].

The PI3K–PKB signaling pathway is involved in many fundamental cellular functions such as proliferation, growth, and survival. The PI3K–PKB signaling pathway is frequently dysregulated by different types of cellular stress stimuli or toxic insults. For example, the activation of PI3K-PKB by upstream activators or amplification of PI3K/PKB genes lead to uncontrolled activation of PKB pathway and it contributes to carcinogenesis [109]. PKB abnormally activated by some protein kinases in leukemia cells. The Fms-like tyrosine kinase-3 (FLT3) receptors within-frame internal tandem duplications (ITD) acts as a upstream for PKB signaling that have been found in about 30% of the patients with acute myeloid leukemia. FLT3-ITD receptors exhibit constitutive tyrosine kinase activity without its ligand binding. Thus, the expression of FLT3-ITD results in relentless activation of PKB and concomitant phosphorylation of FOXO3a in leukemia cells. The phosphorylation of FOXO3a induces its translocation from nucleus to cytoplasm, which in turn leads to suppression of the expression of its target genes *p27*^*Kip1*^ and *Bim* [110]. Similarly, the nucleophosmin–anaplastic lymphoma kinase (NPM-ALK) is a fusion protein kinase which is generated in 30–50% of patients with advanced-stage anaplastic large-cell lymphoma. InBa/F3 cells, the inducible or constitutive expression of NPM-ALK results in concomitant activation of AKT and phosphorylation of FOXO3a, a frequently observed cellular event in anaplastic large-cell lymphoma [111]. A study in mouse model has revealed that Pml deficiency fails to recruit PP2a, PKB phosphatase into PML nuclear bodies, which leads to the accumulation of nuclear phosphor-Akt and nuclear exclusion of FOXO3a. This results in progression of tumorigenesis process in the prostate [112].

The phosphatase and tensin homologue deleted on chromosome 10 (PTEN) is a dual function lipid and protein phosphatase, which was originally identified as a tumor-suppressor. PTEN negatively regulates the PI3K-PKB pathway by dephosphorylation of PI(3,4,5)P3 and down-regulation of PI3K activity. The inactivation of PTEN due to mutations is observed in many primary tumors, such as thyroid, prostate, uterus and breast [109]. The mutation or loss of PTEN activity results in aberrant activation of PKB signaling and nuclear export of FOXO3a during carcinogenesis. In experimental studies found that FOXO3a in PTEN-negative tumors result in cell cycle arrest and apoptosis [3]. Thus, the inactivation of FOXO3a by deregulation of its upstream phosphokinases is crucial for the nuclear export of FOXO3a and acceleration of carcinogenesis. Taken together, these studies strongly suggest that the imbalance between kinases and phosphatases can significantly affect the cellular processes through inhibiting FOXO3a activity, and the alteration of these kinases and phosphatases may cause the dysregulation of FOXO3a leading to carcinogenesis.

#### Effectors of FOXO3a deregulation

Multiple mechanisms have been associated with FOXO3a dysregulation and carcinogenesis due to the fact that it governs many genes involved in apoptosis (such as *Bim*, *Noxa*, *Puma*, *FasL* and *TRAIL*) and cell proliferation (including *p21*, *p27*, *p130*, *Cyclin G2* and *GADD45*) [113]. Our previous study demonstrated that FOXO3a binds to the promoter region of miR-21 and suppresses its promoter activity in human neuroblastoma cells. Fas ligand, a pro-apoptotic factor, is a downstream target of miR-21. Foxo3a inhibits miR-21 transcriptionally which results in to the up-regulation of Fas ligand, and hence initiate the apoptosis [114]. The transcriptional repressor MXI1-SRα is a direct target of FOXO3a, which mediates the repression of MYC activity by FOXO3a [115]. These results indicate that FOXO3a dysregulation contributes to carcinogenesis through directly regulating its target genes expression, and/or affecting its downstream effectors, such as MXI1-SRα.

#### FOXO3a coordinately works with other transcription factors in cancer

FOXO3a has the ability to suppress cancer cell proliferation by down-regulating the expression of several ER-relates genes, which are involved in cell cycle progression. The direct interaction of FOXO3a with ER-α and ER-β proteins causes inhibition of 17β-estradiol (E2)-dependent ER gene transcriptional activities. In ER-positive breast cancer MCF-7 cells, the overexpression of FOXO3a up-regulates the expression of the cyclin-dependent kinase inhibitors (including *p21*^*Cip1*^, *p27*^*Kip1*^, and *p57*^*Kip2*^), which results in the repression of the growth and survival of MCF-7 cells [17]. Molecular studies show that there are several structural and functional similarities between p53 and FOXO3a. Both p53 and FOXO3a control cell cycle progression and DNA damage repair, and both of them can be post-translationally modified by acetylation and phosphorylation. They have regulate a range of genes in common. Thus, there is functional cross talk between these two transcription factors. p53 promotes the expression of SGK, while SGK phosphorylates and inhibits FOXO3a. On the other hand, FOXO3a relieves p53-mediated repression of SIRT1 expression, which, in turn, deacetylates p53 [116]. The transcription factor, RUNX3, is a candidate tumor suppressor that mediates apoptosis and cell growth inhibition in gastric epithelial cells that interacts with FOXO3a and this complex activates Bim to induce apoptosis [60]. FOXO3a also cooperate with other members of forkhead-box transcription factors. For example, FOXO3a interacts with FOXM1 in breast cancer cells and they regulate ERα gene transcription [117]. There is a mutual regulatory mechanisms exist between FOXO3a and other FOX members. In glioblastoma brain tumor cells, SMAD3 is activated by transforming growth factor-β (TGF β) and that forms a complex with FOXO3a to induce the expression of growth inhibitory gene such as *p21*^*Cip1*^, while FOXG1 binds to FOXO3a-SMAD3 complex and blocks *p21*^*Cip1*^ expression [118]. In this context, the potential interaction between different FOX proteins make more complications in understanding the effects of FOX proteins on tumorigenesis. In other way, their interaction provide a point of integration for divergent signaling pathways that could be utilized for the effective therapy.

## FOXO3a as biomarker and therapeutic target in cancer

Currently, due to the physiological and anatomical features of tumor, it is difficult to observe obvious early symptoms of patients, leading to a large number of patients diagnosed at an advanced stage. Therefore, valuable biomarkers for early diagnosis and prognosis of cancer are required in clinical practice. FOXO3a has recently emerged as a potential biomarker for the diagnosis, prognosis and treatment of multiple malignant tumors. For example, FOXO3a expression is identified as a cancer-initiating cells biomarker in Hodgkin’s lymphoma [119]. Many studies showed that FOXO3a expression acts as a prognostic biomarker in multiple cancers [94, 98, 120–127]. Interestingly, overexpression of FOXO3a is associated with poor prognosis in triple-negative breast cancer [120], hepatocellular carcinoma [121], glioblastoma [94] and gastric cancer [122] patients, whereas low expression of FOXO3a is associated with poor prognosis in glioma [126] and ovarian cancer [127] patients. The expression of phosphorylated FOXO3a is also identified as a prognostic biomarker in ovarian cancer [128] and acute myeloid leukemia [129]. The nuclear localization of FOXO3a is demonstrated as a prognostic biomarker in luminal-like breast cancer [130]. In addition, the subcellular localization of FOXO3a is identified as a biomarker for predicting response to the chemotherapy and radiotherapy in cervical carcinoma, breast cancer and esophageal cancer [131, 132]. Although the potential value of FOXO3a as a biomarker has been established in small-scale studies, it is difficult to validate it in large cohorts of patients with cancer. Therefore, further large-scale studies on patient populations are required to confirm the utility of FOXO3a as a biomarker in cancer.

FOXO3a has become a potential target of chemotherapeutic drugs due to its central role in in carcinogenesis. Many chemical and pharmacological agents targeting FOXO3a have been tested in clinical as well as experimental settings. FOXO3 is an indirect target of BMS-345541 (a highly selective IKK inhibitor) in T-cell acute lymphoblastic leukemia (T-ALL) in which the expression of *p21*^*Cip1*^ is up-regulation by increased nuclear translocation of FOXO3a after treatment with BMS-345541. This process is independent of PKB and ERK 1/2 signaling, which indicates that the loss of FOXO3a tumor suppressor function could be mainly due to overactivation of IKK [133]. In BCR-ABL-positive chronic myeloid leukaemia cell lines, STI571 (also called imatinib or Glivec), an inhibitor of BCR-ABL oncoprotein, increases FOXO3a mediated apoptosis by triggering FOXO3a dependent cell cycle arrest and Bim expression [134]. Epigallocatechin-3-gallate (EGCG), the major constituent of green tea, can induce apoptosis by targeting FOXO3a in pancreatic carcinoma [135] and breast carcinoma cells [136]. FOXO3a is also an indirect target of many anticancer agents including paclitaxel [137], cisplatin [138], imatinib [139] and lidamycin [140] in breast cancer cells. All these compounds activate FOXO3a by decreasing PKB activity. However, Paclitaxel also enhances JNK activity, which targets both FOXO3a and 14–3-3 proteins. JNK regulates the activity or stability of FOXO3a by phosphorylation, and this phosphorylation event additionally reduces its interaction with 14–3-3 proteins, which results in the nuclear export of FOXO3a.

The PI3K-PKB pathway is a major downstream signaling pathway of epidermal growth factor receptor (EGFR), which is a crucial cell surface receptor involved in cancer cell proliferation. Thus, the inhibition of EGFR by chemotherapeutic drugs (trastuzumab, lapatinib, afatinib, cetuximab, gefitinib and neratinib) provide a novel and valuable therapeutic strategy for treating breast, colon, prostate, ovarian, lung and head and neck cancers [141, 142] by replenishing the activity of FOXO3a through inhibition of PI3K-PKB. *BNIP3L* is a pro-apoptotic gene, which is required for chemosensitization of cancer cells. This gene is one of the targets of FOXO3a. In breast cancer cell lines, the blockade of EGFR by antibodies or small-molecule inhibitors induces nuclear translocation of FOXO3a and promotes the expression of *BNIP3L* gene, which consequently results in apoptotic death of breast cancer cells [143]. Knockdown of FOXO3a also promotes the response to cetuximab treatment in colorectal cancer [144]. These findings indicate that FOXO3a could be a crucial target of small-molecule EGFR inhibitors, and its activity also increases chemosensitivity of cancer cells to agents such as lapatinib. In agreement with this, the activation of FOXO3a by other anticancer agents also sensitize cancer cells with resistance to apoptosis. For instance, FOXO3a transcriptional activity and its target gene *Bim* expression level is increased in Saos2 (a p53-null osteosarcoma cell line) upon ionizing radiation, which indicates that FOXO3a is a crucial effector of radiation-inducing apoptosis [100]. However, there is a drawback in therapeutically targeting FOXO3a for some type of cancers. *IGFR1* and *PI3KCA* have been identified as target genes of FOXO3a in a colon carcinoma cell line [115], which indicates that FOXO3a may activate PI3K–PKB signaling pathway by multiple mechanisms and it could contribute to drug resistance in colon cancer. However, the majority of studies have revealed that the activation of FOXO3a is highly associated with apoptotic pathway in tumor cells.

FOXO3a activity is directly regulated by a large number of miRNAs. This indicates that the screening or synthesis of novel chemotherapeutic drugs targeting these miRNAs may also be a valuable strategy to treat cancer. Although valuable progress has been made in FOXO3a-based therapeutics for cancer, the most important challenges such as the detailed mechanism of FOXO3a in sensitivity and resistance of chemotherapeutic drugs remain to be solved before its translation in to clinic.

## Conclusions

FOXO3a is a core regulator of multiple physiological and pathological processes by directly inducing or mediating the expression of genes associated with cell proliferation, growth and survival. The deregulation of FOXO3a signaling significantly contributes to the development and progression of many disorders, including cancer. There is a complicated cross-talk between FOXO3a and other key signaling pathways (such as p53 and ER) involved in carcinogenesis. Therefore, FOXO3a is a valuable therapeutic target for a wide range of cancers. The unique role of FOXO3a in the carcinogenesis is that certain tissues offers exciting possibility for cancer-tissue-specific therapeutic strategies. Current studies have shown that FOXO3a targeted chemotherapy has lower toxicity in normal tissues compared with tumor tissues. In chemotherapy-resistant breast cancer cell lines, FOXO3a activation is vital for sensitizing cells to chemotherapeutic agents. ERα is a critical regulator in breast cancer development and it is an efficient target for endocrine therapy [145]. The expression of ERα is considered as a marker for favorable prognosis and the level of functional ERα plays a key role in a successful endocrine treatment for breast cancer [146]. It is well documented that FOXO3a and FOXM1 regulate the expression of ERα [117]. Thus, FOXO3a could be a critical factor in determining the sensitivity and resistance of endocrine treatment. The PI3K-PKB signaling pathway is a relatively stable signaling pathway, which is not commonly mutated in cancers. Therefore, it is a promising strategy to identify novel inhibitors of FOXO3a for future anti-cancer drug design by targeting a downstream node of the PI3K-PKB pathway. As FOXO3a requires the recruitment of co-activators or suppressor for its activity or its inactivation, the therapeutic targeting of the coactivators or corepressors of FOXO3a could also be another way to manipulate FOXO3a functions in cancer cells. This strategy, along with therapeutic manipulation of PTM of FOXO3a would help to avoid the potential side effects in long term due to total inhibition of FOXO3a, which is required for normal cell functions. Given the fact that FOXO3a network is complex and considering its crosstalk with other transcription factors, the influence of FOXO3a in carcinogenesis need to be further investigated in order to develop an efficient FOXO3a based therapeutic strategies. The clinical applications of FOXO3a are potentially promising to limit the progression of human cancers in the future.

## Abbreviations

3′ UTR: 3′-untranslated region
AD: alzheimer’s Alzheimer’s disease
Aβ: AD amyloidAmyloid-β peptide
CARM1: coactivatorCoactivator-associated arginine methyltransferase 1
CBP: CREB-binding protein
CHOP: C/EBP homologous protein
DR: death Death receptor
EGCG: epigallocatechinEpigallocatechin-3-gallate
EGFR: epidermal Epidermal growth factor receptor
ERK: extracellular Extracellular signal-regulated kinase
ERα: estrogen Estrogen receptor α
FKH: forkhead Forkhead winged helix-turn-helix
FKHRL1: forehead Forehead in rhabdomyosarcoma-like 1
FLT3: fmsFms-like tyrosine kinase-3
FOX: forkhead Forkhead box
FOXO: forkhead Forkhead box class O
IBP: interferon Interferon regulatory factor-4 binding protein
IKKβ: IκB kinase isoform β
ITD: internal Internal tandem duplications
LANA2: latency Latency associated nuclear antigen 2
LBD: lewy Lewy body dementia
miRNA: microRNA
MST1: macrophage Macrophage stimulating 1
NES: nuclear Nuclear export sequence
NFκB: nuclear Nuclear factor κB
NLS: nuclear Nuclear localization sequence
NPM-ALK: nucleophosminNucleophosmin–anaplastic lymphoma; kinase
PD: pParkinson'’s disease
PI3K: phosphoinositolPhosphoinositol-3-kinase
PKB: protein Protein kinase B
POF: premature Premature ovarian failure
PTEN: phosphatase Phosphatase and tensin homologue deleted on chromosome 10
PTMs: postPost-translational modifications
SGK: serumSerum-and glucocorticoid-inducible kinases
TAD: transactivation Transactivation domain
T-ALL: t-cell acute lymphoblastic leukemia
TGF β: transforming Transforming growth factor-β

## References

[1] Benayoun BA, Caburet S, Veitia RA (2011). Forkhead transcription factors: key players in health and disease. Trends Genet.

[2] Wang Y, Zhou Y, Graves DT (2014). FOXO transcription factors: their clinical significance and regulation. Biomed Res Int.

[3] Myatt SS, Lam EW (2007). The emerging roles of forkhead box (fox) proteins in cancer. Nat Rev Cancer.

[4] Zhang W, Duan N, Song T, Li Z, Zhang C, Chen X (2017). The emerging roles of Forkhead box (FOX) proteins in osteosarcoma. J Cancer.

[5] Carlsson P, Mahlapuu M (2002). Forkhead transcription factors: key players in development and metabolism. Dev Biol.

[6] Hannenhalli S, Kaestner KH (2009). The evolution of fox genes and their role in development and disease. Nat Rev Genet.

[7] Murtaza G, Khan AK, Rashid R, Muneer S, Hasan SMF, Chen J (2017). FOXO transcriptional factors and long-term living. Oxidative Med Cell Longev.

[8] Weigel D, Jurgens G, Kuttner F, Seifert E, Jackle H (1989). The homeotic gene fork head encodes a nuclear protein and is expressed in the terminal regions of the Drosophila embryo. Cell.

[9] Tikhanovich I, Cox J, Weinman SA (2013). Forkhead box class O transcription factors in liver function and disease. J Gastroenterol Hepatol.

[10] Gomes AR, Zhao F, Lam EW (2013). Role and regulation of the forkhead transcription factors FOXO3a and FOXM1 in carcinogenesis and drug resistance. Chin J Cancer.

[11] Maiese K (2015). FoxO proteins in the nervous system. Anal Cell Pathol (Amst).

[12] van der Vos KE, Gomez-Puerto C, Coffer PJ (2012). Regulation of autophagy by Forkhead box (FOX) O transcription factors. Adv Biol Regul.

[13] Anderson MJ, Viars CS, Czekay S, Cavenee WK, Arden KC (1998). Cloning and characterization of three human forkhead genes that comprise an FKHR-like gene subfamily. Genomics.

[14] Zanella F, Rosado A, Garcia B, Carnero A, Link W (2008). Chemical genetic analysis of FOXO nuclear-cytoplasmic shuttling by using image-based cell screening. Chembiochem.

[15] Rinner O, Mueller LN, Hubalek M, Muller M, Gstaiger M, Aebersold R (2007). An integrated mass spectrometric and computational framework for the analysis of protein interaction networks. Nat Biotechnol.

[16] Nielsen MD, Luo X, Biteau B, Syverson K, Jasper H (2008). 14-3-3 epsilon antagonizes FoxO to control growth, apoptosis and longevity in Drosophila. Aging Cell.

[17] Zou Y, Tsai WB, Cheng CJ, Hsu C, Chung YM, Li PC, Lin SH, Hu MC (2008). Forkhead box transcription factor FOXO3a suppresses estrogen-dependent breast cancer cell proliferation and tumorigenesis. Breast Cancer Res.

[18] Wang F, Marshall CB, Yamamoto K, Li GY, Plevin MJ, You H, Mak TW, Ikura M (2008). Biochemical and structural characterization of an intramolecular interaction in FOXO3a and its binding with p53. J Mol Biol.

[19] Van Der Heide LP, Hoekman MF, Smidt MP (2004). The ins and outs of FoxO shuttling: mechanisms of FoxO translocation and transcriptional regulation. Biochem J.

[20] Ambros V (2004). The functions of animal microRNAs. Nature.

[21] Ryan BM (2017). microRNAs in Cancer susceptibility. Adv Cancer Res.

[22] Willingham AT, Gingeras TR (2006). TUF love for "junk" DNA. Cell.

[23] Kong W, He L, Coppola M, Guo J, Esposito NN, Coppola D, Cheng JQ (2010). MicroRNA-155 regulates cell survival, growth, and chemosensitivity by targeting FOXO3a in breast cancer. J Biol Chem.

[24] Ji WG, Zhang XD, Sun XD, Wang XQ, Chang BP, Zhang MZ (2014). miRNA-155 modulates the malignant biological characteristics of NK/T-cell lymphoma cells by targeting FOXO3a gene. J Huazhong Univ Sci Technolog Med Sci.

[25] Wu H, Huang T, Ying L, Han C, Li D, Xu Y, Zhang M, Mou S, Dong Z (2016). MiR-155 is involved in renal ischemia-reperfusion injury via direct targeting of FoxO3a and regulating renal tubular cell Pyroptosis. Cell Physiol Biochem.

[26] Ling N, Gu J, Lei Z, Li M, Zhao J, Zhang HT, Li X (2013). microRNA-155 regulates cell proliferation and invasion by targeting FOXO3a in glioma. Oncol Rep.

[27] Liao WW, Zhang C, Liu FR, Wang WJ (2018). Effects of miR-155 on proliferation and apoptosis by regulating FoxO3a/BIM in liver Cancer Cell line HCCLM3. Eur Rev Med Pharmacol Sci.

[28] Wong HK, Veremeyko T, Patel N, Lemere CA, Walsh DM, Esau C, Vanderburg C, Krichevsky AM (2013). De-repression of FOXO3a death axis by microRNA-132 and -212 causes neuronal apoptosis in Alzheimer's disease. Hum Mol Genet.

[29] Kim HY, Kwon HY, Ha Thi HT, Lee HJ, Kim GI, Hahm KB, Hong S (2016). MicroRNA-132 and microRNA-223 control positive feedback circuit by regulating FOXO3a in inflammatory bowel disease. J Gastroenterol Hepatol.

[30] Ge YF, Sun J, Jin CJ, Cao BQ, Jiang ZF, Shao JF (2013). AntagomiR-27a targets FOXO3a in glioblastoma and suppresses U87 cell growth in vitro and in vivo. Asian Pac J Cancer Prev.

[31] Sun L, Zhao M, Wang Y, Liu A, Lv M, Li Y, Yang X, Wu Z (2017). Neuroprotective effects of miR-27a against traumatic brain injury via suppressing FoxO3a-mediated neuronal autophagy. Biochem Biophys Res Commun.

[32] Lin H, Dai T, Xiong H, Zhao X, Chen X, Yu C, Li J, Wang X, Song L (2010). Unregulated miR-96 induces cell proliferation in human breast cancer by downregulating transcriptional factor FOXO3a. PLoS One.

[33] Nho RS, Im J, Ho YY, Hergert P (2014). MicroRNA-96 inhibits FoxO3a function in IPF fibroblasts on type I collagen matrix. Am J Physiol Lung Cell Mol Physiol.

[34] Li X, Du N, Zhang Q, Li J, Chen X, Liu X, Hu Y, Qin W, Shen N, Xu C (2014). MicroRNA-30d regulates cardiomyocyte pyroptosis by directly targeting foxo3a in diabetic cardiomyopathy. Cell Death Dis.

[35] Hudson MB, Rahnert JA, Zheng B, Woodworth-Hobbs ME, Franch HA, Price SR (2014). miR-182 attenuates atrophy-related gene expression by targeting FoxO3 in skeletal muscle. Am J Physiol Cell Physiol.

[36] Fu Q, Du Y, Yang C, Zhang D, Zhang N, Liu X, Cho WC, Yang Y (2016). An oncogenic role of miR-592 in tumorigenesis of human colorectal cancer by targeting Forkhead box O3A (FoxO3A). Expert Opin Ther Targets.

[37] Qiu X, Dou Y (2017). miR-1307 promotes the proliferation of prostate cancer by targeting FOXO3A. Biomed Pharmacother.

[38] Guerit D, Brondello JM, Chuchana P, Philipot D, Toupet K, Bony C, Jorgensen C, Noel D (2014). FOXO3A regulation by miRNA-29a controls chondrogenic differentiation of mesenchymal stem cells and cartilage formation. Stem Cells Dev.

[39] Cai J, Fang L, Huang Y, Li R, Yuan J, Yang Y, Zhu X, Chen B, Wu J, Li M (2013). miR-205 targets PTEN and PHLPP2 to augment AKT signaling and drive malignant phenotypes in non-small cell lung cancer. Cancer Res.

[40] Sanphui P, Biswas SC (2013). FoxO3a is activated and executes neuron death via Bim in response to beta-amyloid. Cell Death Dis.

[41] Wang X, Hu S, Liu L (2017). Phosphorylation and acetylation modifications of FOXO3a: independently or synergistically?. Oncol Lett.

[42] Calnan DR, Brunet A (2008). The FoxO code. Oncogene.

[43] Daitoku H, Sakamaki J, Fukamizu A (2011). Regulation of FoxO transcription factors by acetylation and protein-protein interactions. Biochim Biophys Acta.

[44] Plas DR, Thompson CB (2003). Akt activation promotes degradation of tuberin and FOXO3a via the proteasome. J Biol Chem.

[45] Yang JY, Zong CS, Xia W, Yamaguchi H, Ding Q, Xie X, Lang JY, Lai CC, Chang CJ, Huang WC (2008). ERK promotes tumorigenesis by inhibiting FOXO3a via MDM2-mediated degradation. Nat Cell Biol.

[46] Finnberg N, El-Deiry WS (2004). Activating FOXO3a, NF-kappaB and p53 by targeting IKKs: an effective multi-faceted targeting of the tumor-cell phenotype?. Cancer Biol Ther.

[47] Luo J, Liang A, Liang M, Xia R, Rizvi Y, Wang Y, Cheng J (2016). Serum glucocorticoid-regulated kinase 1 blocks CKD-induced muscle wasting via inactivation of FoxO3a and Smad2/3. J Am Soc Nephrol.

[48] Lu J, Zhang R, Hong H, Yang Z, Sun D, Sun S, Guo X, Ye J, Li Z, Liu P (2016). The poly(ADP-ribosyl)ation of FoxO3 mediated by PARP1 participates in isoproterenol-induced cardiac hypertrophy. Biochim Biophys Acta.

[49] Ho KK, McGuire VA, Koo CY, Muir KW, de Olano N, Maifoshie E, Kelly DJ, McGovern UB, Monteiro LJ, Gomes AR (2012). Phosphorylation of FOXO3a on Ser-7 by p38 promotes its nuclear localization in response to doxorubicin. J Biol Chem.

[50] Lehtinen MK, Yuan Z, Boag PR, Yang Y, Villen J, Becker EB, DiBacco S, de la Iglesia N, Gygi S, Blackwell TK (2006). A conserved MST-FOXO signaling pathway mediates oxidative-stress responses and extends life span. Cell.

[51] Sanchez AM, Csibi A, Raibon A, Cornille K, Gay S, Bernardi H, Candau R (2012). AMPK promotes skeletal muscle autophagy through activation of forkhead FoxO3a and interaction with Ulk1. J Cell Biochem.

[52] Giannakou ME, Partridge L (2004). The interaction between FOXO and SIRT1: tipping the balance towards survival. Trends Cell Biol.

[53] Wang F, Nguyen M, Qin FX, Tong Q (2007). SIRT2 deacetylates FOXO3a in response to oxidative stress and caloric restriction. Aging Cell.

[54] Ghosh AP, Klocke BJ, Ballestas ME, Roth KA (2012). CHOP potentially co-operates with FOXO3a in neuronal cells to regulate PUMA and BIM expression in response to ER stress. PLoS One.

[55] Chandramohan V, Mineva ND, Burke B, Jeay S, Wu M, Shen J, Yang W, Hann SR, Sonenshein GE (2008). C-Myc represses FOXO3a-mediated transcription of the gene encoding the p27(Kip1) cyclin dependent kinase inhibitor. J Cell Biochem.

[56] Munoz-Fontela C, Marcos-Villar L, Gallego P, Arroyo J, Da Costa M, Pomeranz KM, Lam EW, Rivas C (2007). Latent protein LANA2 from Kaposi's sarcoma-associated herpesvirus interacts with 14-3-3 proteins and inhibits FOXO3a transcription factor. J Virol.

[57] Li J, Du W, Maynard S, Andreassen PR, Pang Q (2010). Oxidative stress-specific interaction between FANCD2 and FOXO3a. Blood.

[58] Miyaguchi Y, Tsuchiya K, Sakamoto K (2009). P53 negatively regulates the transcriptional activity of FOXO3a under oxidative stress. Cell Biol Int.

[59] Singh A, Ye M, Bucur O, Zhu S, Tanya Santos M, Rabinovitz I, Wei W, Gao D, Hahn WC, Khosravi-Far R (2010). Protein phosphatase 2A reactivates FOXO3a through a dynamic interplay with 14-3-3 and AKT. Mol Biol Cell.

[60] Yamamura Y, Lee WL, Inoue K, Ida H, Ito Y (2006). RUNX3 cooperates with FoxO3a to induce apoptosis in gastric cancer cells. J Biol Chem.

[61] Jacobs KM, Pennington JD, Bisht KS, Aykin-Burns N, Kim HS, Mishra M, Sun L, Nguyen P, Ahn BH, Leclerc J (2008). SIRT3 interacts with the daf-16 homolog FOXO3a in the mitochondria, as well as increases FOXO3a dependent gene expression. Int J Biol Sci.

[62] Chen YF, Pandey S, Day CH, Chen YF, Jiang AZ, Ho TJ, Chen RJ, PadmaViswanadha V, Kuo WW, Huang CY (2017). Synergistic effect of HIF-1alpha and FoxO3a trigger cardiomyocyte apoptosis under hyperglycemic ischemia condition. J Cell Physiol.

[63] McClelland Descalzo DL, Satoorian TS, Walker LM, Sparks NR, Pulyanina PY, Zur Nieden NI (2016). Glucose-induced oxidative stress reduces proliferation in embryonic stem cells via FOXO3A/beta-catenin-dependent transcription of p21(cip1). Stem Cell Reports.

[64] McGowan SE, McCoy DM (2013). Platelet-derived growth factor-a regulates lung fibroblast S-phase entry through p27(kip1) and FoxO3a. Respir Res.

[65] Joseph J, Ametepe ES, Haribabu N, Agbayani G, Krishnan L, Blais A, Sad S (2016). Inhibition of ROS and upregulation of inflammatory cytokines by FoxO3a promotes survival against Salmonella typhimurium. Nat Commun.

[66] Fluteau A, Ince PG, Minett T, Matthews FE, Brayne C, Garwood CJ, Ratcliffe LE, Morgan S, Heath PR, Shaw PJ (2015). The nuclear retention of transcription factor FOXO3a correlates with a DNA damage response and increased glutamine synthetase expression by astrocytes suggesting a neuroprotective role in the ageing brain. Neurosci Lett.

[67] Wang X, Chen WR, Xing D (2012). A pathway from JNK through decreased ERK and Akt activities for FOXO3a nuclear translocation in response to UV irradiation. J Cell Physiol.

[68] Lim SW, Jin L, Luo K, Jin J, Shin YJ, Hong SY, Yang CW (2017). Klotho enhances FoxO3-mediated manganese superoxide dismutase expression by negatively regulating PI3K/AKT pathway during tacrolimus-induced oxidative stress. Cell Death Dis.

[69] Wang X, Meng L, Zhao L, Wang Z, Liu H, Liu G, Guan G (2017). Resveratrol ameliorates hyperglycemia-induced renal tubular oxidative stress damage via modulating the SIRT1/FOXO3a pathway. Diabetes Res Clin Pract.

[70] Willcox BJ, Donlon TA, He Q, Chen R, Grove JS, Yano K, Masaki KH, Willcox DC, Rodriguez B, Curb JD (2008). FOXO3A genotype is strongly associated with human longevity. Proc Natl Acad Sci U S A.

[71] Zhao J, Brault JJ, Schild A, Cao P, Sandri M, Schiaffino S, Lecker SH, Goldberg AL (2007). FoxO3 coordinately activates protein degradation by the autophagic/lysosomal and proteasomal pathways in atrophying muscle cells. Cell Metab.

[72] Matrone A, Grossi V, Chiacchiera F, Fina E, Cappellari M, Caringella AM, Di Naro E, Loverro G, Simone C (2010). p38alpha is required for ovarian cancer cell metabolism and survival. Int J Gynecol Cancer.

[73] Hu MC, Lee DF, Xia W, Golfman LS, Ou-Yang F, Yang JY, Zou Y, Bao S, Hanada N, Saso H (2004). IkappaB kinase promotes tumorigenesis through inhibition of forkhead FOXO3a. Cell.

[74] Qin W, Zhao W, Ho L, Wang J, Walsh K, Gandy S, Pasinetti GM (2008). Regulation of forkhead transcription factor FoxO3a contributes to calorie restriction-induced prevention of Alzheimer's disease-type amyloid neuropathology and spatial memory deterioration. Ann N Y Acad Sci.

[75] Su B, Liu H, Wang X, Chen SG, Siedlak SL, Kondo E, Choi R, Takeda A, Castellani RJ, Perry G (2009). Ectopic localization of FOXO3a protein in Lewy bodies in Lewy body dementia and Parkinson's disease. Mol Neurodegener.

[76] Skurk C, Izumiya Y, Maatz H, Razeghi P, Shiojima I, Sandri M, Sato K, Zeng L, Schiekofer S, Pimentel D (2005). The FOXO3a transcription factor regulates cardiac myocyte size downstream of AKT signaling. J Biol Chem.

[77] Tan WQ, Wang K, Lv DY, Li PF (2008). Foxo3a inhibits cardiomyocyte hypertrophy through transactivating catalase. J Biol Chem.

[78] Chaanine AH, Jeong D, Liang L, Chemaly ER, Fish K, Gordon RE, Hajjar RJ (2012). JNK modulates FOXO3a for the expression of the mitochondrial death and mitophagy marker BNIP3 in pathological hypertrophy and in heart failure. Cell Death Dis.

[79] Hauck L, Harms C, Grothe D, An J, Gertz K, Kronenberg G, Dietz R, Endres M, von Harsdorf R (2007). Critical role for FoxO3a-dependent regulation of p21CIP1/WAF1 in response to statin signaling in cardiac myocytes. Circ Res.

[80] Galasso G, De Rosa R, Piscione F, Iaccarino G, Vosa C, Sorriento D, Piccolo R, Rapacciuolo A, Walsh K, Chiariello M (2010). Myocardial expression of FOXO3a-Atrogin-1 pathway in human heart failure. Eur J Heart Fail.

[81] Sandri M, Sandri C, Gilbert A, Skurk C, Calabria E, Picard A, Walsh K, Schiaffino S, Lecker SH, Goldberg AL (2004). Foxo transcription factors induce the atrophy-related ubiquitin ligase atrogin-1 and cause skeletal muscle atrophy. Cell.

[82] Rathbone CR, Booth FW, Lees SJ (2008). FoxO3a preferentially induces p27Kip1 expression while impairing muscle precursor cell-cycle progression. Muscle Nerve.

[83] Senf SM, Dodd SL, McClung JM, Judge AR (2008). Hsp70 overexpression inhibits NF-kappaB and Foxo3a transcriptional activities and prevents skeletal muscle atrophy. FASEB J.

[84] Clavel S, Siffroi-Fernandez S, Coldefy AS, Boulukos K, Pisani DF, Derijard B (2010). Regulation of the intracellular localization of Foxo3a by stress-activated protein kinase signaling pathways in skeletal muscle cells. Mol Cell Biol.

[85] Allard D, Figg N, Bennett MR, Littlewood TD (2008). Akt regulates the survival of vascular smooth muscle cells via inhibition of FoxO3a and GSK3. J Biol Chem.

[86] Vinci G, Christin-Maitre S, Pasquier M, Bouchard P, Fellous M, Veitia RA (2008). FOXO3a variants in patients with premature ovarian failure. Clin Endocrinol.

[87] Zhang S, Huan W, Wei H, Shi J, Fan J, Zhao J, Shen A, Teng H (2013). FOXO3a/p27kip1 expression and essential role after acute spinal cord injury in adult rat. J Cell Biochem.

[88] Mojsilovic-Petrovic J, Nedelsky N, Boccitto M, Mano I, Georgiades SN, Zhou W, Liu Y, Neve RL, Taylor JP, Driscoll M (2009). FOXO3a is broadly neuroprotective in vitro and in vivo against insults implicated in motor neuron diseases. J Neurosci.

[89] Zhang X, Zhuang T, Liang Z, Li L, Xue M, Liu J, Liang H (2017). Breast cancer suppression by aplysin is associated with inhibition of PI3K/AKT/FOXO3a pathway. Oncotarget.

[90] Shukla S, Bhaskaran N, Maclennan GT, Gupta S (2013). Deregulation of FoxO3a accelerates prostate cancer progression in TRAMP mice. Prostate.

[91] Thepot S, Lainey E, Cluzeau T, Sebert M, Leroy C, Ades L, Tailler M, Galluzzi L, Baran-Marszak F, Roudot H (2011). Hypomethylating agents reactivate FOXO3A in acute myeloid leukemia. Cell Cycle.

[92] Shoeb M, Ramana KV, Srivastava SK (2013). Aldose reductase inhibition enhances TRAIL-induced human colon cancer cell apoptosis through AKT/FOXO3a-dependent upregulation of death receptors. Free Radic Biol Med.

[93] Yang YC, Tang YA, Shieh JM, Lin RK, Hsu HS, Wang YC (2014). DNMT3B overexpression by deregulation of FOXO3a-mediated transcription repression and MDM2 overexpression in lung cancer. J Thorac Oncol.

[94] Qian Z, Ren L, Wu D, Yang X, Zhou Z, Nie Q, Jiang G, Xue S, Weng W, Qiu Y (2017). Overexpression of FoxO3a is associated with glioblastoma progression and predicts poor patient prognosis. Int J Cancer.

[95] Marlow LA, von Roemeling CA, Cooper SJ, Zhang Y, Rohl SD, Arora S, Gonzales IM, Azorsa DO, Reddi HV, Tun HW (2012). Foxo3a drives proliferation in anaplastic thyroid carcinoma through transcriptional regulation of cyclin A1: a paradigm shift that impacts current therapeutic strategies. J Cell Sci.

[96] Blake DC, Mikse OR, Freeman WM, Herzog CR (2010). FOXO3a elicits a pro-apoptotic transcription program and cellular response to human lung carcinogen nicotine-derived nitrosaminoketone (NNK). Lung Cancer.

[97] Fang L, Wang H, Zhou L, Yu D (2011). Akt-FOXO3a signaling axis dysregulation in human oral squamous cell carcinoma and potent efficacy of FOXO3a-targeted gene therapy. Oral Oncol.

[98] Shou Z, Lin L, Liang J, Li JL, Chen HY (2012). Expression and prognosis of FOXO3a and HIF-1alpha in nasopharyngeal carcinoma. J Cancer Res Clin Oncol.

[99] Shiota M, Song Y, Yokomizo A, Kiyoshima K, Tada Y, Uchino H, Uchiumi T, Inokuchi J, Oda Y, Kuroiwa K (2010). Foxo3a suppression of urothelial cancer invasiveness through Twist1, Y-box-binding protein 1, and E-cadherin regulation. Clin Cancer Res.

[100] Yang JY, Xia W, Hu MC (2006). ionizing radiation activates expression of FOXO3a, Fas ligand, and Bim, and induces cell apoptosis. Int J Oncol.

[101] Yu C, Zhang Z, Liao W, Zhao X, Liu L, Wu Y, Liu Z, Li Y, Zhong Y, Chen K (2012). The tumor-suppressor gene Nkx2.8 suppresses bladder cancer proliferation through upregulation of FOXO3a and inhibition of the MEK/ERK signaling pathway. Carcinogenesis.

[102] Santo EE, Stroeken P, Sluis PV, Koster J, Versteeg R, Westerhout EM (2013). FOXO3a is a major target of inactivation by PI3K/AKT signaling in aggressive neuroblastoma. Cancer Res.

[103] Ding Q, Chen Y, Zhang Q, Guo Y, Huang Z, Dai L, Cao S (2015). 8bromo7methoxychrysin induces apoptosis by regulating Akt/FOXO3a pathway in cisplatinsensitive and resistant ovarian cancer cells. Mol Med Rep.

[104] Guo JP, Tian W, Shu S, Xin Y, Shou C, Cheng JQ (2013). IKBKE phosphorylation and inhibition of FOXO3a: a mechanism of IKBKE oncogenic function. PLoS One.

[105] Wang YQ, Cao Q, Wang F, Huang LY, Sang TT, Liu F, Chen SY (2015). SIRT1 protects against oxidative stress-induced endothelial progenitor cells apoptosis by inhibiting FOXO3a via FOXO3a ubiquitination and degradation. J Cell Physiol.

[106] Bader AG, Kang S, Zhao L, Vogt PK (2005). Oncogenic PI3K deregulates transcription and translation. Nat Rev Cancer.

[107] Heasley LE, Han SY (2006). JNK regulation of oncogenesis. Mol Cells.

[108] Perkins ND (2007). Integrating cell-signalling pathways with NF-kappaB and IKK function. Nat Rev Mol Cell Biol.

[109] Osaki M, Oshimura M, Ito H (2004). PI3K-Akt pathway: its functions and alterations in human cancer. Apoptosis.

[110] Scheijen B, Ngo HT, Kang H, Griffin JD (2004). FLT3 receptors with internal tandem duplications promote cell viability and proliferation by signaling through Foxo proteins. Oncogene.

[111] Gu TL, Tothova Z, Scheijen B, Griffin JD, Gilliland DG, Sternberg DW (2004). NPM-ALK fusion kinase of anaplastic large-cell lymphoma regulates survival and proliferative signaling through modulation of FOXO3a. Blood.

[112] Chae HK, Siberio-Perez DY, Kim J, Go Y, Eddaoudi M, Matzger AJ, O'Keeffe M, Yaghi OM (2004). A route to high surface area, porosity and inclusion of large molecules in crystals. Nature.

[113] van Grevenynghe J, Cubas RA, DaFonseca S, Metcalf T, Tremblay CL, Trautmann L, Sekaly RP, Schatzle J, Haddad EK (2012). Foxo3a: an integrator of immune dysfunction during HIV infection. Cytokine Growth Factor Rev.

[114] Wang K, Li PF (2010). Foxo3a regulates apoptosis by negatively targeting miR-21. J Biol Chem.

[115] Delpuech O, Griffiths B, East P, Essafi A, Lam EW, Burgering B, Downward J, Schulze A (2007). Induction of Mxi1-SR alpha by FOXO3a contributes to repression of Myc-dependent gene expression. Mol Cell Biol.

[116] You H, Mak TW (2005). Crosstalk between p53 and FOXO transcription factors. Cell Cycle.

[117] Madureira PA, Varshochi R, Constantinidou D, Francis RE, Coombes RC, Yao KM, Lam EW (2006). The Forkhead box M1 protein regulates the transcription of the estrogen receptor alpha in breast cancer cells. J Biol Chem.

[118] Seoane J, Le HV, Shen L, Anderson SA, Massague J (2004). Integration of Smad and forkhead pathways in the control of neuroepithelial and glioblastoma cell proliferation. Cell.

[119] Ikeda J, Tian T, Wang Y, Hori Y, Honma K, Wada N, Morii E (2013). Expression of FoxO3a in clinical cases of malignant lymphoma. Pathol Res Pract.

[120] Rehman A, Kim Y, Kim H, Sim J, Ahn H, Chung MS, Shin SJ, Jang K. FOXO3a expression is associated with lymph node metastasis and poor disease-free survival in triple-negative breast cancer. J Clin Pathol. 2018; 10.1136/jclinpath-2018-205052 29588373

[121] Ahn H, Kim H, Abdul R, Kim Y, Sim J, Choi D, Paik SS, Shin SJ, Kim DH, Jang K (2018). Overexpression of Forkhead box O3a and its association with aggressive phenotypes and poor prognosis in human hepatocellular carcinoma. Am J Clin Pathol.

[122] Yu S, Yu Y, Sun Y, Wang X, Luo R, Zhao N, Zhang W, Li Q, Cui Y, Wang Y (2015). Activation of FOXO3a suggests good prognosis of patients with radically resected gastric cancer. Int J Clin Exp Pathol.

[123] Liu HB, Gao XX, Zhang Q, Liu J, Cui Y, Zhu Y, Liu YF (2015). Expression and prognostic implications of FOXO3a and Ki67 in lung adenocarcinomas. Asian Pac J Cancer Prev.

[124] Jiang Y, Zou L, Lu WQ, Zhang Y, Shen AG (2013). Foxo3a expression is a prognostic marker in breast cancer. PLoS One.

[125] Lu M, Zhao Y, Xu F, Wang Y, Xiang J, Chen D (2012). The expression and prognosis of FOXO3a and Skp2 in human ovarian cancer. Med Oncol.

[126] Shi J, Zhang L, Shen A, Zhang J, Wang Y, Zhao Y, Zou L, Ke Q, He F, Wang P (2010). Clinical and biological significance of forkhead class box O 3a expression in glioma: mediation of glioma malignancy by transcriptional regulation of p27kip1. J Neuro-Oncol.

[127] Fei M, Zhao Y, Wang Y, Lu M, Cheng C, Huang X, Zhang D, Lu J, He S, Shen A (2009). Low expression of Foxo3a is associated with poor prognosis in ovarian cancer patients. Cancer Investig.

[128] Lu M, Xiang J, Xu F, Wang Y, Yin Y, Chen D (2012). The expression and significance of pThr32-FOXO3a in human ovarian cancer. Med Oncol.

[129] Kornblau SM, Singh N, Qiu Y, Chen W, Zhang N, Coombes KR (2010). Highly phosphorylated FOXO3A is an adverse prognostic factor in acute myeloid leukemia. Clin Cancer Res.

[130] Habashy HO, Rakha EA, Aleskandarany M, Ahmed MA, Green AR, Ellis IO, Powe DG (2011). FOXO3a nuclear localisation is associated with good prognosis in luminal-like breast cancer. Breast Cancer Res Treat.

[131] Kim HJ, Lee SY, Kim CY, Kim YH, Ju W, Kim SC (2017). Subcellular localization of FOXO3a as a potential biomarker of response to combined treatment with inhibitors of PI3K and autophagy in PIK3CA-mutant cancer cells. Oncotarget.

[132] Chen MF, Fang FM, Lu CH, Lu MS, Chen WC, Lee KD, Lin PY (2008). Significance of nuclear accumulation of Foxo3a in esophageal squamous cell carcinoma. Int J Radiat Oncol Biol Phys.

[133] Buontempo F, Chiarini F, Bressanin D, Tabellini G, Melchionda F, Pession A, Fini M, Neri LM, McCubrey JA, Martelli AM (2012). Activity of the selective IkappaB kinase inhibitor BMS-345541 against T-cell acute lymphoblastic leukemia: involvement of FOXO3a. Cell Cycle.

[134] Kikuchi S, Nagai T, Kunitama M, Kirito K, Ozawa K, Komatsu N (2007). Active FKHRL1 overcomes imatinib resistance in chronic myelogenous leukemia-derived cell lines via the production of tumor necrosis factor-related apoptosis-inducing ligand. Cancer Sci.

[135] Shankar S, Marsh L, Srivastava RK (2013). EGCG inhibits growth of human pancreatic tumors orthotopically implanted in Balb C nude mice through modulation of FKHRL1/FOXO3a and neuropilin. Mol Cell Biochem.

[136] Belguise K, Guo S, Sonenshein GE (2007). Activation of FOXO3a by the green tea polyphenol epigallocatechin-3-gallate induces estrogen receptor alpha expression reversing invasive phenotype of breast cancer cells. Cancer Res.

[137] Khongkow M, Olmos Y, Gong C, Gomes AR, Monteiro LJ, Yague E, Cavaco TB, Khongkow P, Man EP, Laohasinnarong S (2013). SIRT6 modulates paclitaxel and epirubicin resistance and survival in breast cancer. Carcinogenesis.

[138] Wilson MS, Brosens JJ, Schwenen HD, Lam EW (2011). FOXO and FOXM1 in cancer: the FOXO-FOXM1 axis shapes the outcome of cancer chemotherapy. Curr Drug Targets.

[139] Yang JY, Hung MC (2009). A new fork for clinical application: targeting forkhead transcription factors in cancer. Clin Cancer Res.

[140] Yang AJ, Shi WW, Li Y, Wang Z, Shao RG, Li DD, He QY (2009). Role of prosurvival molecules in the action of lidamycin toward human tumor cells. Biomed Environ Sci.

[141] O'Neill F, Madden SF, Clynes M, Crown J, Doolan P, Aherne ST, O'Connor R (2013). A gene expression profile indicative of early stage HER2 targeted therapy response. Mol Cancer.

[142] Reid A, Vidal L, Shaw H, de Bono J (2007). Dual inhibition of ErbB1 (EGFR/HER1) and ErbB2 (HER2/neu). Eur J Cancer.

[143] Real PJ, Benito A, Cuevas J, Berciano MT, de Juan A, Coffer P, Gomez-Roman J, Lafarga M, Lopez-Vega JM, Fernandez-Luna JL (2005). Blockade of epidermal growth factor receptors chemosensitizes breast cancer cells through up-regulation of Bnip3L. Cancer Res.

[144] Yu Y, Peng K, Li H, Zhuang R, Wang Y, Li W, Yu S, Liang L, Xu X, Liu T (2018). SP1 upregulated FoxO3a promotes tumor progression in colorectal cancer. Oncol Rep.

[145] Keen JC, Davidson NE (2003). The biology of breast carcinoma. Cancer.

[146] Ali S, Coombes RC (2002). Endocrine-responsive breast cancer and strategies for combating resistance. Nat Rev Cancer.

